# Supplementary material for: Gender-specific hearing loss in German adults aged 18 to 84 years compared to US-American and current European studies
Source: PLoS One. 2020 Apr 23;15(4):e0231632. doi: 10.1371/journal.pone.0231632 (PMC7179866; doi:10.1371/journal.pone.0231632)
Supplement: Attachment — Submitted filename: Response_to_Reviewers.docx [file pone.0231632.s001.docx]

Responses to the Reviewer

# 1 **Abstract**

### Reviewer

Rather than refer to “international standards such as ISO 1999” which readers such as myself may be unfamiliar with could the authors point out the direction of the gender difference: rather than simply saying that the gender gap was smaller in Germany compared to the US.

### Response

Thank you for the suggestion. We modified the abstract in the paragraphs ‘Introduction’ and ‘Results’. Nevertheless, we consider the standardization an important topic, particularly ISO 1999, and kept the corresponding arguments in the introduction.

# 2 **Introduction**

### Reviewer

The authors should give the study location for the study by Zhan et al (REF 3) to match the details given for the other studies. Given the reference to gender gap I was surprised that the authors did not refer to this in their research hypotheses.

### Response

We included the study location for the study of Zhan et al., as suggested. The basic concept that modifiable factors might play an important role in the development of HI in the elderly is certainly neither far-fetched nor singular, especially not from the perspective of social epidemiology. However, we agree that Zhan et al. provided strong evidence for that in their comprehensive analysis. Therefore, we cited a key statement of Zhan et al. just before posing our research hypotheses (at the end of the introduction).

# 3 **Methodology**

## **Grouped data**

### Reviewer

I did not understand the sentence (line 206-207) which states that “Generally, measurement data were regarded as grouped data”. The authors should state clearly what they mean without readers having to look-up the reference.

### Response

We rephrased this section and briefly explained the assumption for this method of percentile calculation and its main advantage. Specifically, the insertion reads as follows:

*Generally, measurement data were regarded as grouped data using the algorithm implemented in SPSS 25.0 [20]. As outlined by Dobie [21] the grouped data approach is less sensitive to small distributional changes, because it assumes an even distribution of the ‘real’ HTL within the 5-dB step size used in audiometry.*

## **Between-group differences**

### Reviewer

I also found it very surprising that the authors relied on non-overlapping confidence intervals to consider between group differences as statistically significant. This is a conservative approach: would a formal test have been better? In the results the authors state that the “gender differences failed statistical significance…” is this because of the use of non-overlapping CIs?

### Response

Admittedly, non-overlap of 95%-CI is a conservative approach in the evaluation of between-group differences. We adopted this criterion because it has its merits and is easy to trace from the results given in the corresponding table. However, we additionally included formal significance testing based on CHI² statistics in the revised manuscript.

Specifically, the insertion in the section ‘Materials and Methods’ reads as follows:

*95% confidence intervals (CI) are given for all prevalence estimates and the hearing-aid adoption rate, to allow for evaluating between-group differences. Additionally, Pearson Chi² test was performed (2-sided, significance level 0.05).*

In the ‘Results’ section, the following paragraph was inserted:

*The estimates’ 95%-CI for females and males overlapped in all comparisons. Based on CHI² statistics, however, gender differences reached significance in the 60 and 80 year age groups as well as in the total sample for HI_WHO_ with p < 0.01, and in the 80 year age group for hearing-aid adoption with p = 0.023. All other gender-related comparisons failed the significance criterion.*

# 4 **Results**

## **Mean vs Median**

### Reviewer

The authors refer to the mean for the results presented in Table 1: this is confusing. I assume they meant medians.

### Response

We meant means, but the wording was unclear. We have reworded the corresponding sentences.

To clarify in detail: As stated in the manuscript (Methods and Materials), Nienke Homans kindly provided percentile estimates for this analysis. However, these were conventional percentile estimates that are appropriate for estimating PTA, but not for evaluating small differences of single HTL. We therefore referred to the published results of Homans et al. (2017), which were mean HTL for 5-year age bands, separately for the left and right ears. We’ve tried to get as close as possible to these published Dutch results, and also calculated mean HTL for 5-year age bands separately for the left (L) and right (R) ears. For the comparison, the L-HTL and R-HTL were averaged separately for each age band, resulting in an age-specific LR-HTL. The range of differences in dB HL reported in the manuscript refers to age-specific LR-HTL difference between the Dutch data and our results at the frequencies 0.25, .5, 1, and 4 kHz averaged across age bands. We reported this result because we wanted to give an order of magnitude of the HTL differences, not only for PTA, but without overloading the manuscript with descriptive details. Therefore, this paragraph was revised as follows (changes are underlined):

*In contrast, the differences of the age-specific mean HTL (left and right ear average, not shown here) between the Dutch and our estimates were smaller for males than for females, and basically limited to 4 kHz and frequencies below 2 kHz. In terms of dB, HTL differences at 0.25, 0.5, 1, and 4 kHz accounted for 3 to 6 dB in females but only 2 to 3 dB in males on average. Age-specific HTL differences at 2 and 8 kHz were ≤ 1 dB, and thus well below the uncertainty due to equipment and calibration.*

## **Labeling**

### Reviewer

Perhaps frequency in Table 1 would be better labelled as referred to in the Methods section: i.e. 0.25, 0.5, 1, 2, 3, 4, 6, and 8

### Response

Done.

# 5 **Discussion**

## **Ethnicity**

### Reviewer

I applaud the wishes of the authors to avoid making ‘vague references’ to factors such as social welfare provision for differences in hearing levels between countries. However I was wondering whether the comparisons between the US and German estimates would be strengthened by a consideration of race/ethnicity for the former. The authors briefly mention ethnicity as a limitation of their study. However, I would have liked a little more on the discussion of ethnicity. Have there been any German studies that shed light on ethnic variations in hearing?

### Response

Ethnicity has never been addressed in earlier German hearing studies. The ongoing large longitudinal health study, the German National Cohort (NAKO), is expected to recruit a sample that possibly allows for a differentiated analysis with regard to ethnicity. NAKO basically uses the same speech audiometric test in noise in a subsample that has been used in the UK Biobank study (Dawes et al., 2014, now cited). However, sampling and analysis will take some more time. Against this background, we’ve found no compelling reason for discussing in detail possible ethnic HTL variations in this comparative analysis. However, we inserted a paragraph with regard to the contradicting findings in the US American and the UK Biobank data. This insertion reads as follows:

*However, ethnic affiliations were not recorded, neither in the present study nor any earlier hearing study in Germany. Hence, there is no basis for ethnicity-specific comparisons to analyses of US data where hearing loss was found to be more pronounced in white Americans than, particularly, in black Americans of the same gender and age [25, 57]. However, this finding was not confirmed by Dawes et al. (2014) for the large UK Biobank database [58]. In contrast to the US results, odds for hearing impairment measured with a speech recognition test in noise increased with ethnic minority backgrounds, including in British black persons.*

## **Policy and Public Health**

### Reviewer

I also wonder whether the discussion is short on policy and public-health implications. Although age-related hearing impairment seems to be less smaller in Europe what initiatives are – and could be put – in place to continue to reduce age-related hearing loss.

### Response

This study aimed at a comparative analysis of published results on age-related hearing loss. We discussed possible reasons that might partially explain the observed differences, but without being able to verify or even quantify their effectiveness. In our opinion, the latter would be necessary to substantiate further discussions of policy or public-health implications in this manuscript, though not generally.

Nevertheless, we have taken up the issue of the different health care systems. Still trying to avoid ‘vague references’, we assume that systemic under-coverage in health care could contribute to the observed differences by means of non-diagnosed and non-treated pathologies that are considered independent risk factors for hearing loss.

Specifically, the insertion reads as follows:

*“The health systems of the countries included in the comparison differ markedly. More importantly, there is a general difference between the European countries and the USA. The European countries essentially pursue systems regulated by the state or by social partnerships and often sustained by compulsory insurance, which in principle guarantees health care for every citizen. In contrast, in the US, health care is mostly based on private arrangements, and governmental health care regulations reach only a comparatively small proportion of the population. According to the official statistics for the year 2011, 17.7% of the US citizens aged 18 and above had no health-insurance coverage at any time during the year [38]. As showed by Freeman et al. (2008) in their systematic review, however, health insurance had substantial effects on the use of health services and health outcomes [39]. Thus widespread pathologies that might impact hearing may be less frequently undiagnosed and receive more frequent, continuous medical treatment in Europe than in the US. For example, there is evidence that overall, males are at a higher risk for diabetic diseases [42], and that diabetic diseases should be regarded as independent risk factors for hearing loss [40, 41]. Thus, systemic under-coverage in health care could have indirectly contributed to the comparatively large gender gap in the results from the US results.”*

## **Hearing-aid adoption**

### Reviewer

For example, although the authors present estimates for hearing aid adoption they do not discuss who pays for the costs associated with hearing aid use?

### Response

The topic ‘Hearing-aid adoption’ was thoroughly examined and discussed in another article based on the same data and published by the same authors (Holube et al., 2019). We referred to this article, which is freely available in English language, and have added more details on this article in the revised manuscript. Nevertheless, we still think that the information on hearing-aid adoption rates is reasonable when reporting and discussing the prevalence of hearing impairment using two competing criteria (WHO and GBD). As regards the coverage of costs in Germany, fitting of HA in the basic category is covered by health insurance, provided that specific hearing-loss criteria are met. This subject was also addressed in the above-mentioned article and is, in our opinion, beyond the scope of the present contribution.

## **Extended discussion**

### Insertion independent from review

After submission we became aware of a study researching hearing abilities in veterans and non-veterans. Wilson et al. (2010) found male veterans and non-veterans equally likely to have hearing loss. This finding contradicts the concept that a higher percentage of veterans in the US society might also have contributed to the higher HI found in males in the US than the European studies. We therefore additionally considered this study in the discussion and included to following paragraph:

“*However, it is questionable whether the higher proportion of men who have done military service contributed to a higher HI prevalence in US males. Wilson et al. (2010) used various measures for comparing the hearing abilities and performance of 999 male veterans and 590 male non-veterans in the EHLS data [43]. They found veterans and non-veterans equally likely to have a hearing loss*.”

# 6 **Figures**

### Reviewer

I would like the figures to have higher resolution in a final version: plus a consideration of using black and white rather than colour.

### Response

Graphics in EPS-format were uploaded. If required, we can provide the figures with high resolution in almost any other format. We refrained from switching to black and white graphics, because Figure 3 definitely requires colors and symbols. However, we assume that the colors chosen in Figures 1 and 2 should still be distinguishable when printed in black and white.
